# Thyroid nodule rupture after radiofrequency ablation: case report and literature review

**DOI:** 10.3389/fendo.2024.1355383

**Published:** 2024-04-02

**Authors:** Tatiana Ferraro, Sameeha Sajid, Steven P. Hodak, Chelsey K. Baldwin

**Affiliations:** ^1^ Division of Otolaryngology-Head and Neck Surgery, The George Washington University School of Medicine and Health Sciences, Washington, DC, United States; ^2^ Department of Endocrinology, The George Washington University School of Medicine & Health Sciences, Washington, DC, United States; ^3^ Department of Medicine, Diabetes and Endocrinology Section, New York University School of Medicine, New York, NY, United States

**Keywords:** radiofrequency ablation, thyroid nodule rupture, minimally invasive techniques, thermal ablation, thyroid nodule

## Abstract

**Purpose:**

Radiofrequency ablation (RFA) is an effective and safe modality for the treatment of thyroid nodules. Nodule rupture is a major complication of RFA. There is little known on the natural history of nodule rupture due to a lack of clinical experience and no consensus on its management. A comprehensive review of nodule rupture presentation, diagnosis, and management is needed.

**Methods:**

We report a case of nodule rupture and conduct a literature review. A total of 33 patients experiencing nodule rupture after RFA were included, and their clinical presentation, management, and outcomes were collected and analyzed.

**Results:**

Nodule rupture presents with acute swelling (90.3%) and pain (77.4%) within 7 months of RFA procedure, most commonly due to disruption of the anterior thyroid capsule (87%), and can be diagnosed with ultrasonography. Most ruptures can be managed conservatively, exemplified by our reported case. There are no reported cases of long-term sequalae.

**Conclusion:**

Nodule rupture is the second most common major complication of RFA. Based on the available evidence, we propose a treatment algorithm for nodule rupture and recommendations for future data collection to address gaps in our understanding of rupture etiology and effective management.

## 1 Introduction

Radiofrequency ablation (RFA) is a minimally invasive technique that is an effective and safe treatment modality for benign symptomatic thyroid nodules. Average nodule volume reduction of 67.7%–84.8% at 12 months following RFA has been reported (1–4) leading to symptomatic and cosmetic improvement as measured by patient health-related quality of life scores (3). However, RFA is associated with a number of possible adverse events. These include minor complications such as local pressure, pain, vasovagal reaction, cough, hematoma, vomiting, and skin burns, and major complications such as both transient and permanent voice changes, hypo/hyperthyroidism, nerve plexus injury, and nodule rupture (5).

Following voice injury, nodule rupture is the second most common major complication associated with RFA occurring in 0.2%–2.5% of cases (1, 3). Nodule rupture usually requires a significant escalation of follow-up intensity and sequelae, may range from local discomfort to formation of an abscess with mass effect on the trachea requiring urgent surgical management (3, 6). Nodule rupture is defined by the disruption of the thyroid capsule with extravasation of nodule contents into the extra thyroidal space (5). The pathophysiology of nodule rupture is unknown. Reported management strategies include conservative measures of pain control and antibiotic therapy, and invasive measures including surgical evacuation and thyroid lobectomy (1, 2, 6). Currently, there is a paucity of data on nodule rupture including consensus on etiology and management despite the increasing number of clinicians performing RFA procedure for thyroid structural disease. In this study, we report our experience with a case of nodule rupture and review the available literature for risk factors, etiology, methods of diagnosis, and management. Finally, we propose a standard of data collection to improve our understanding of this serious complication of RFA.

## 2 Case report

Informed consent was provided for all clinical information and images associated with this publication.

**Figure 1:**
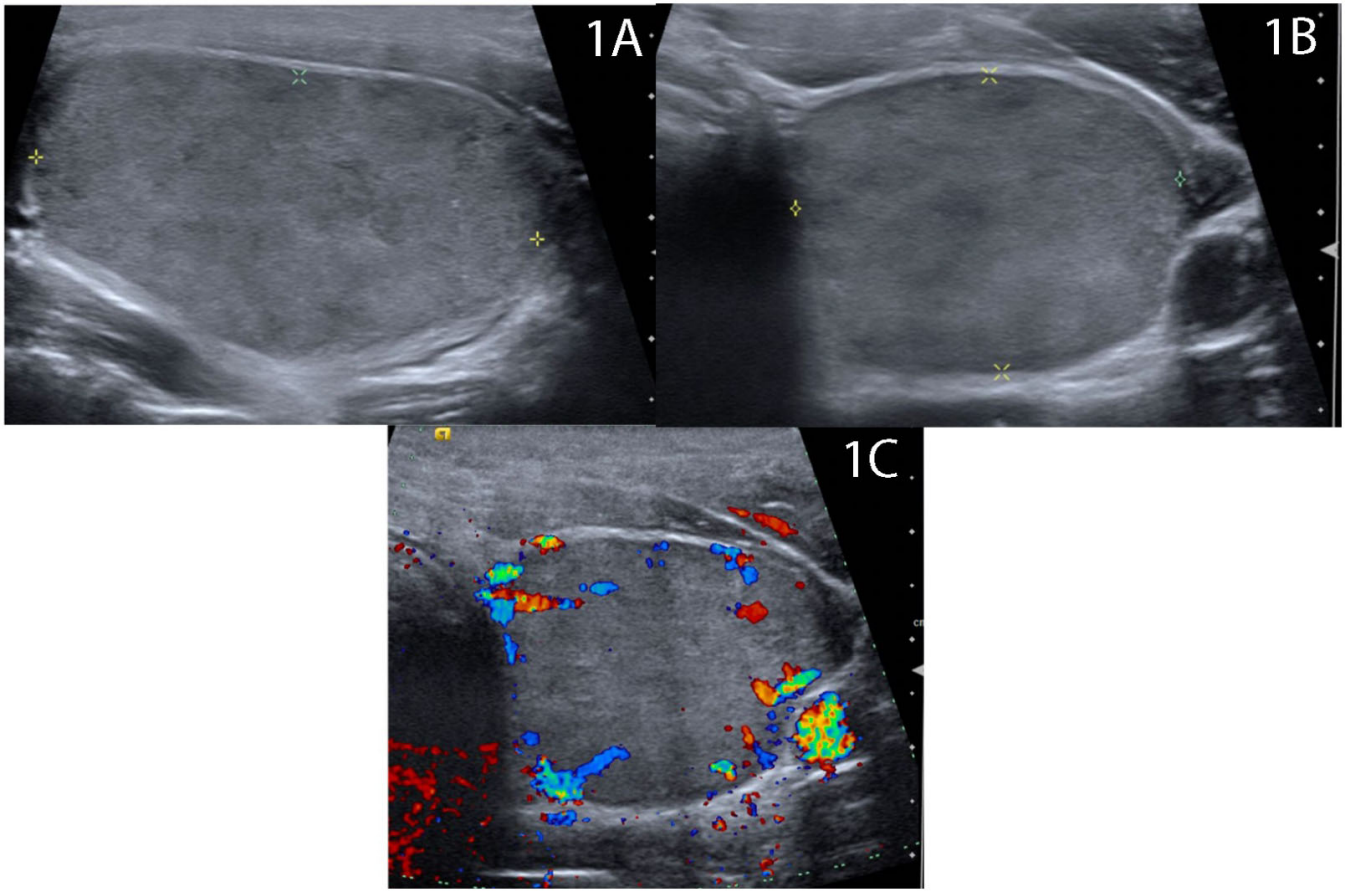
Baseline ultrasound demonstrates 4.2 cm solid homogenous, mildly hypoechoic nodule thyroid nodule replacing the majority of the left thyroid lobe in **(A)** longitude and **(B)** transverse views with **(C)** scant peripheral vascularity.

A 47-year-old man with a history of congenital heart disease presented seeking alternatives to surgical management for a cosmetically bothersome left lobe thyroid nodule associated with compressive symptoms while wearing a buttoned collar. Ultrasound of the thyroid revealed a solid, mildly hypoechoic nodule nearly replacing the left lobe measuring 42 × 23 × 29 mm (14.7 mL) (**Figure 1**). Fine needle aspiration demonstrated benign cytology. The patient was biochemically euthyroid with a baseline TSH of 1.37 mIU/L (0.4–4.1 mIU/L). The patient opted to undergo radiofrequency ablation. On the day of the procedure, the patient was given 1 mg of Xanax for anxiolytic effect, and 1% lidocaine without epinephrine was infiltrated in the skin at the planned needle entry site and around the anterior thyroid capsule to provide local analgesia. A 7-cm-length, 18-ga, 1-cm active tip, internally cooled radiofrequency (RF) electrode (Well-Point RF Electrode, Taewoong Medical, Seoul Korea) was used via the trans-isthmic approach with moving shot technique. Power was initiated at 35 W, and a maximum power of 60 W was used. The patient tolerated the procedure well with only transient pauses for discomfort. Upon completion, ultrasound imaging confirmed typical hypo- and hyperechoic changes consistent with treatment effect throughout the entire volume of the nodule; there was no evidence of immediate complication, and the patient was discharged 60 min after the completion of the case.

**Figure 2:**
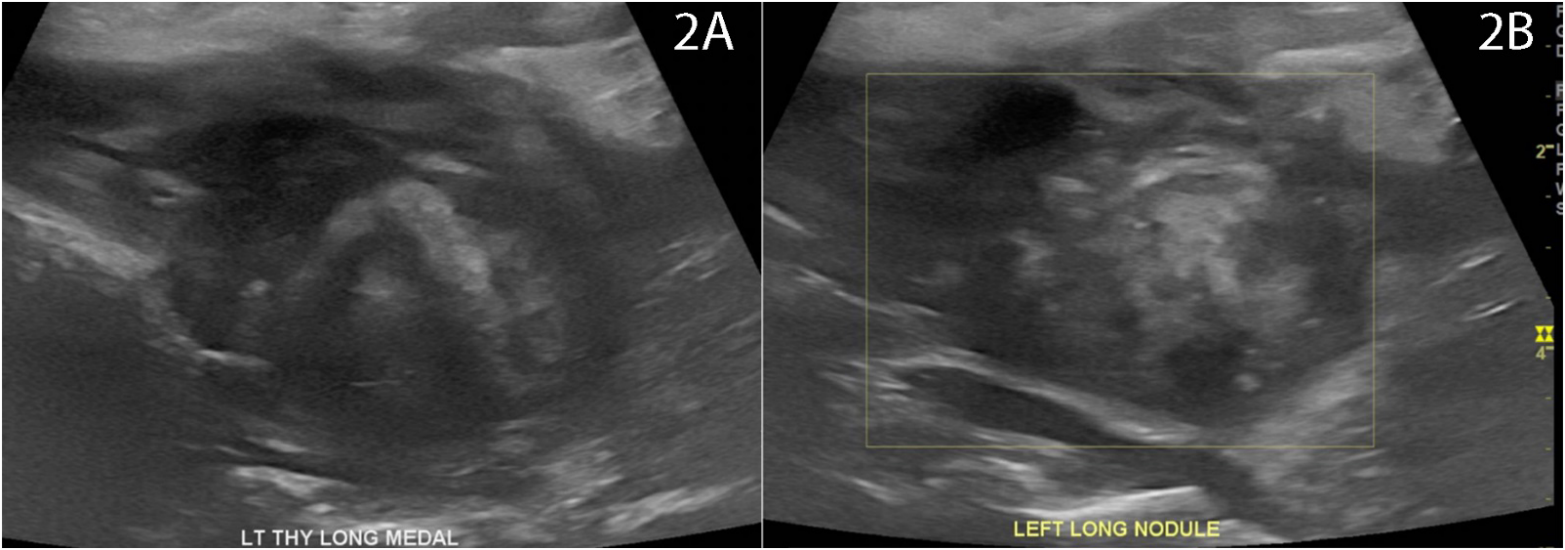
Photograph of patient neck at **(A)** 7 days post-RFA at time of initial complaint of transient redness and discomfort with spontaneous recovery and **(B)** at time of recurrent symptoms of pain, swelling, and redness at 1 month post-RFA prompting CT neck demonstrating findings of nodule rupture.

**Figure 3:**
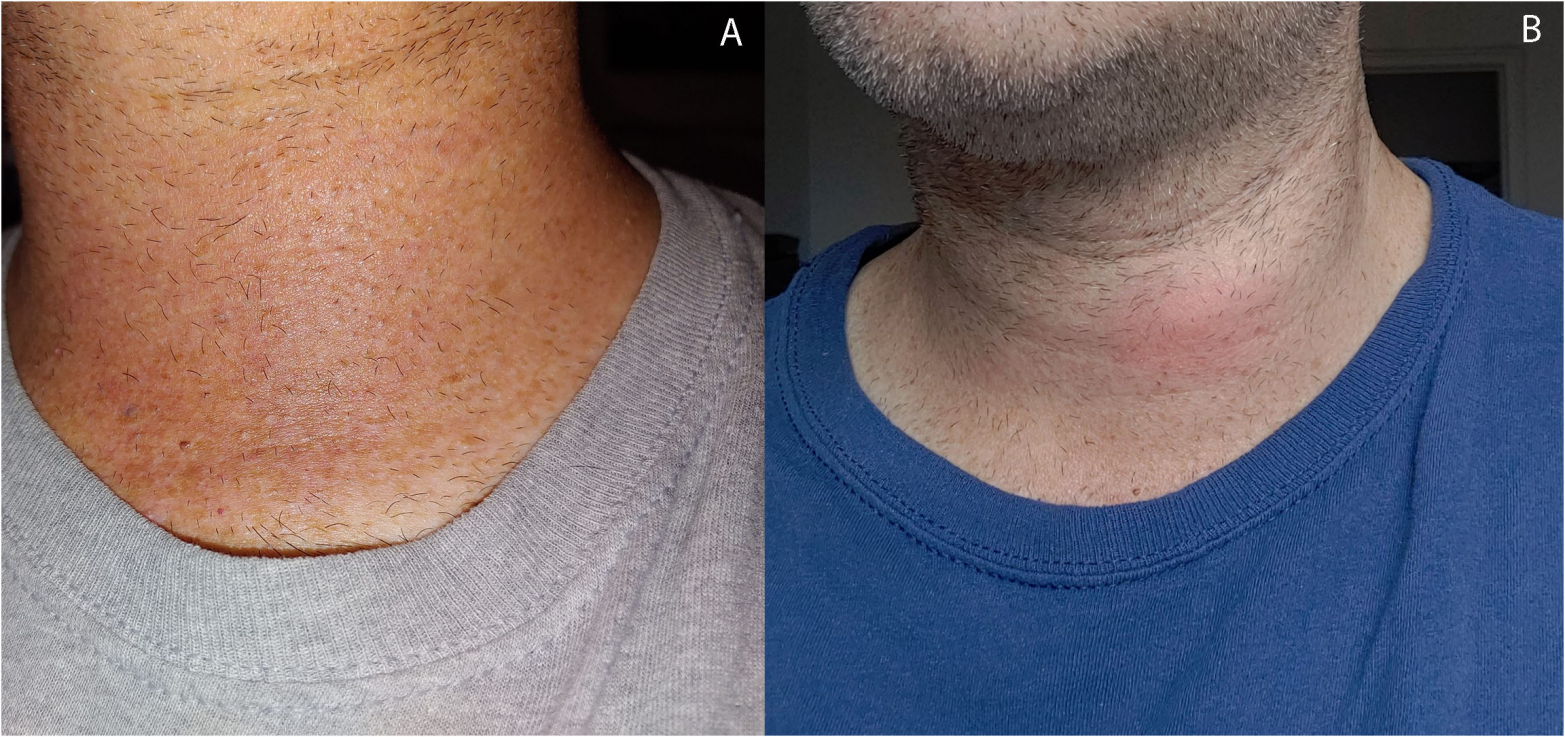
Ultrasound 7 days post-RFA with patient reported symptoms of transient erythema and fullness. Ultrasound without extrathyroidal mass but classic artifacts typical of immediate post-RFA including areas of **(A)** hyperechoic and hypoechoic changes with a non-distinct anterior border **(B)** without evidence of active bleeding.

One week post-RFA, the patient reported transient anterior neck discomfort associated with mild erythema for 24 h, which resolved without intervention (**Figure 2A**). Ultrasound performed within 1 day of complaint demonstrated the continued presence of expected post-treatment changes including a non-distinct anterior border in close proximity to the treatment zone, however, with no evidence of extrathyroidal fluid extravasation (**Figure 3A**) or evidence of active bleeding (**Figure 3B**).

**Figure 4:**
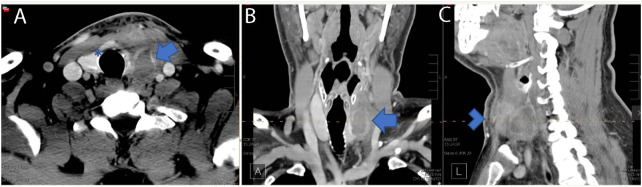
Four weeks post-RFA contrasted neck CT demonstrating a 3.3 cm × 2.1 cm × 3.5 cm (AP × TR × CC) low-density perithyroidal fluid collection with peripheral enhancement (arrow) deep to the sternocleidomastoid muscle with disruption of the anterior capsule (Asterix) associated with edema and reticulation of the overlying subcutaneous tissue and skin (arrowhead) consistent with nodule rupture. Features of the nodule rupture are shown in **(A)** transverse, **(B)** coronal, and **(C)** sagittal planes.

**Figure 5:**
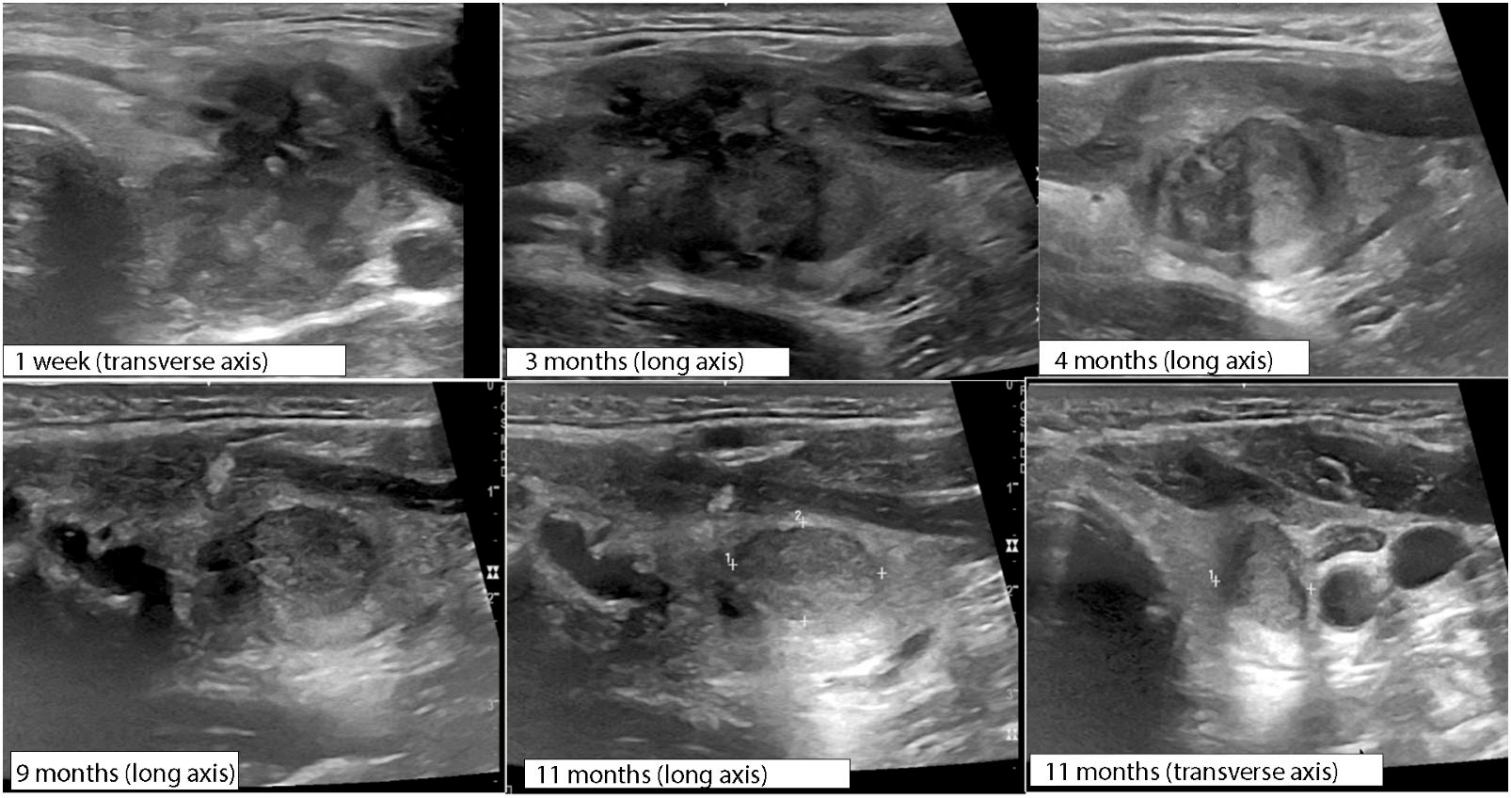
Serial ultrasound changes for 1 year following initial RFA procedure measured at time post-rupture, monitoring nodule rupture resolution.

At 4 weeks post-RFA, the patient reported mild, recurrent 3/10 anterior neck pain associated with redness and swelling (**Figure 2B**). The maximum temperature measured at home was 100.0°F. The patient also reported dental work 2 days prior to recurrent pain. The patient presented to a local facility for evaluation, and contrast-enhanced neck CT showed a 3.3 cm × 2.1 cm × 3.5 cm (AP × TR × CC) low-density perithyroidal fluid collection contiguous with the treated thyroid nodule with peripheral enhancement deep to the sternocleidomastoid muscle associated with edema and reticulation of the overlying subcutaneous tissue and skin (**Figure 4**) consistent with thyroid nodule rupture. Findings were confirmed on subsequent ultrasonography (**Figure 5**). Because of the recent dental procedure and concern for potential infection, Augmentin 500–200 mg daily for 10 days was initiated and surveillance was continued.

The patient reported resolution of pain after 3 days with persistent prominence of the left neck. Over the course of the next 3 months, the patient reported slow resolution of neck fullness. Progress was monitored with ultrasound imaging over the course of 1 year following RFA (**Figure 5**) demonstrating progressive resolution of rupture findings. Ultimately, there was a 93% nodule volume reduction from baseline without development of recurrent pain or thyroid hormone dysfunction [TSH of 1.51 mIU/L (0.4–4.1)] at 1 year post-RFA.

## 3 Literature review

We identified seven studies in the literature that reported a total of 33 cases of nodule rupture after radiofrequency ablation (RFA). Studies predominantly included benign thyroid nodules (BTN) but also included malignant nodules. All cases of nodule rupture occurred in BTN (**Table 1**). Study inclusion criteria included English Language and report of thyroid nodule rupture. Best judgment was used to eliminant redundant cases used in serial publications.

**Table 1:** Characteristics, procedure, management of nodule rupture in eight case reports/series.

Study	Age Mean + range (years)	Gender (% male)	Mean nodule volume (mL)	Rate of rupture/nodules treated	Mean time to onset of symptoms (days)	RUP (min)	Mean total energy used (kcal)	Mean max power of all RFA sessions (W)	Symptoms	Method of diagnosis	Location of nodule rupture	Peri-rupture complications	Outcome
Baek 2012 & Kim 2017 (N=3)	–	–	37.5 (range, 17.4–71.5)	3/875 = 0.34%	34 days (range, 22–50)	–	–	–	Swelling 100% Pain 100%	US 66% US&CT33%	Anterior 100%	Internal Bleeding 66% Abscess 33%	CR 100%
Shin 2011 (N=6)	43 (28–60) years	33% male	22.5 (range, 2.12–57.47)	6/2616 = 0.2%	38.5 days (range, 9–60)	23.7	–	83.2 W (range, 50–130)	Swelling 100% Pain 100%	US Only 33% CT only 17% US&CT 50%	Anterior 100%	–	CR 100%
Che 2015 (N=1)	–	100% male	–	1/200 = 0.5%	7 days	–	–	–	–	–	–	–	CR 2 weeks
Valcalvi 2015 (N=1)	–	–	–	1/40 = 2.5%	26 days	–	–	–	–	–	–	Fasciitis	CR
Chung 2019 (N=12)	40.6 (16–75) years	25% male	17.18 mL (range, 0.19–75.13)	–	50.5 days (range, 11–156)	12.2	–	57.5 W (range, 30–110)	Sudden Swelling 83.3% Pain 83.3% Cough 8.3% Fever 8.3%	US only 83.3% CT only 8.3% US & CT 8.3%	Anterior 75% Posterolateral 16.7% Medial 8.3%	–	CR ~100% (11/11)*
Chen 2021 (N=9)	38.9 (range, 27–48) yrs	22.2% male	73.9 mL (range 7.7–198)	9/818 = 1.1%	51.4 days (range, 19–195)	29.3 (Std dv 13.5)	16.6 kcal (range, 1.5–33.3)	58.8 W (range, 30–70)	Swelling 88.9% Pain 33.3% Erythema 44.4% Fever 11.1%	CT only 33.3% US only 66.7%	Anterior 88.9% Medial 11.1%	–	CR 100%
Our case study (N=1)	47	100% male	14.67 mL	–	21 days	–	–	60 W	Swelling 100% Pain 100% Erythema 100%	CT & US	Anterior 100%	–	CR
All Studies (N=33)	41.5 (16–75)	36.4% male (12/33)	36.57 mL (0.19–198)	0.44% (20/4,549)	48.8 days (7–195)	20.5 min	16.6 kcal	64.9 W	Swelling 90.3% (28/31) Pain 77.4% (24/31) Erythema 16.1% (5/31) Fever 6.5% (2/31) Cough (1/31) =3.2%	US Only 64.5% (20/31) CT Only 16.1% (5/31) US & CT 19.4% (6/31)	Ant 27/31 = 87.1% posterolateral= 2/31 = 6.5% medial= 2/31 = 6.5%	Superimposed infection (abscess or fasciitis) =6.0% (2/33)	CR 100% (32/32)

Abx, antibiotics; HA, hospital admission; I&D, incision and drainage; SD, surgical debridement; NR, not reported; CR, complete recovery; –, data not reported; RUP, ablation time in minutes; of the RFA session immediately prior to rupture; Mean Max Power= the average obtained for maximum power achieved across all RFA sessions; *Chung 2019 study one patient without report of management technique so eliminated from this chart bring n = 11.

## 4 Discussion

### 4.1 Presentation and diagnosis

Nodule rupture is the second most common major complication of radiofrequency ablation occurring at a frequency of 0.21%–2.5% (1, 3). Nodule rupture is not unique to RFA and has been reported following microwave and laser ablation as well (7, 8).

Nodule rupture after RFA typically presents with acute swelling (90.3%), pain (77.4%), and erythema (16.1%) of the anterior neck (**Table 1**). Relatively few patients have fever (6.5%), and only one patient reported cough associated with a medial rupture content compressing the trachea (2). Onset of symptoms ranges from 16 to 195 days post-procedure with an average of 48.8 days (**Table 1**), suggesting that rupture typically presents within the first 2 months following RFA, however should be considered up to 7 months post-procedure (1, 5).

The diagnosis of nodule rupture is confirmed by imaging demonstrating the disruption of the thyroid capsule with nodule contents extravasating into the extrathyroidal space. Extrathyroidal contents most commonly spread within the soft tissue with inflammatory response consistent with phlegmon and less commonly form organized abscess (1). Thyroid ultrasonography is sensitive for the diagnosis of rupture; however, patients presenting to emergency services are more likely to be diagnosed by CT (1). Familiarity with ultrasonographic findings consistent with nodule rupture would obviate the need for unnecessary diagnostic procedures such as CT or aspiration and expedite correct diagnosis (2).

**Figure 6:**
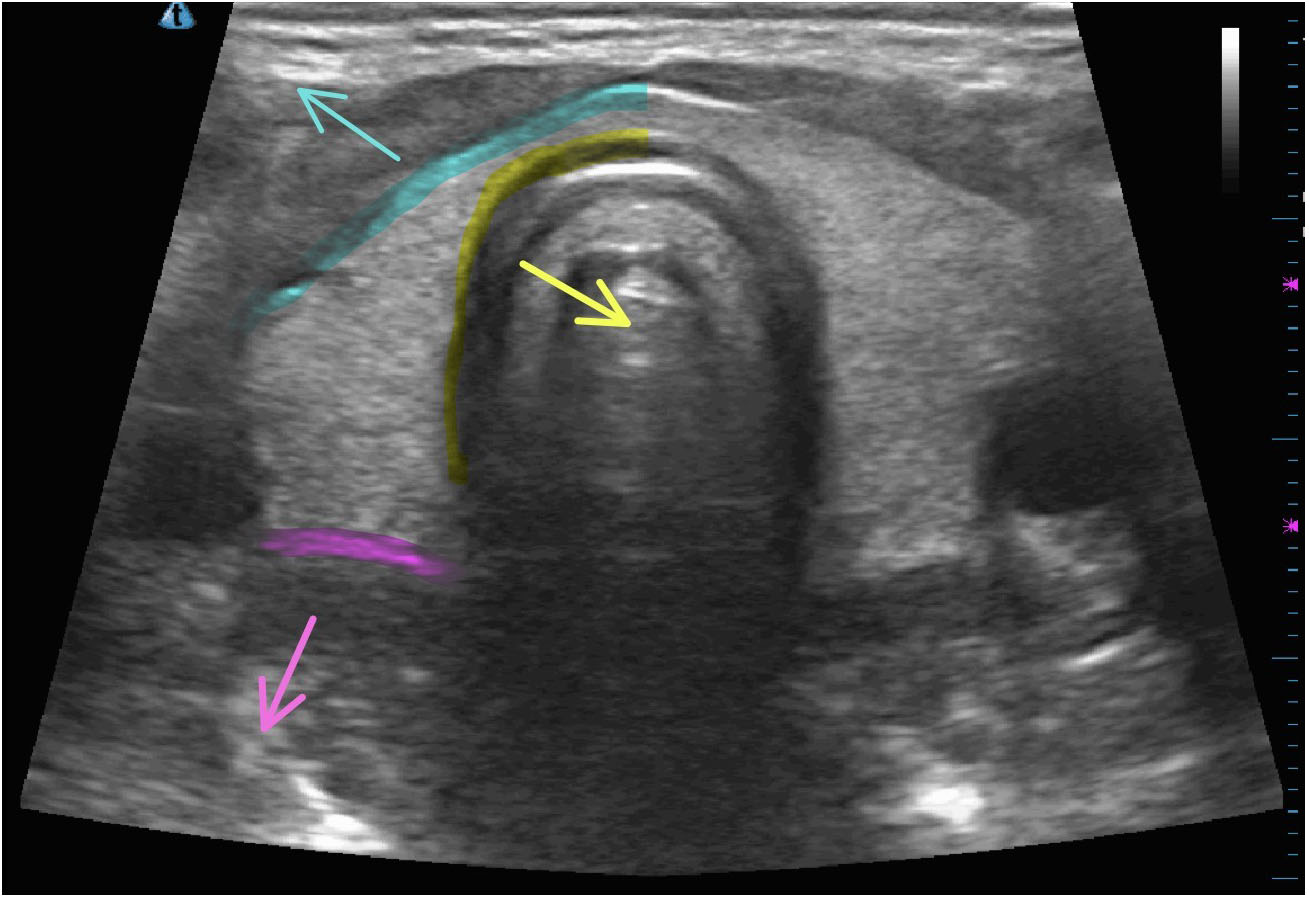
Locations of nodule rupture on normal thyroid ultrasonographic imaging with arrows indicating anterior rupture (blue), medial rupture (yellow), and posterolateral rupture (pink).

**Figure 7:**
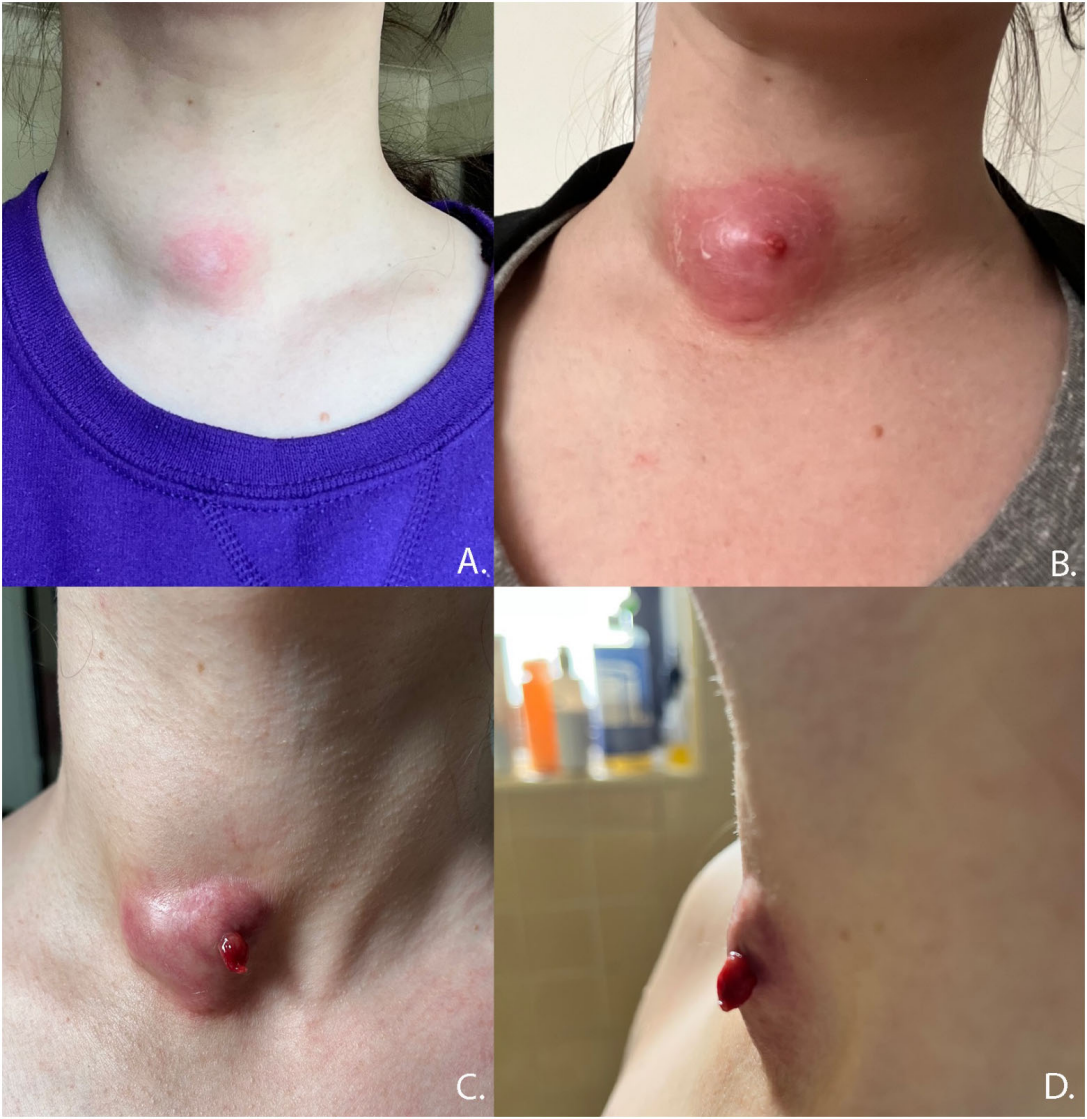
Progression of anterior nodule rupture with sinus track development. **(A)** 60 days post-RFA procedure, **(B)** 67 days post RFA, **(C, D)** 82 days post-RFA.

Nodule ruptures can be classified by the location of thyroid capsule disruption: anterior, posterolateral, and medial (**Figure 6**) (2). Anterior ruptures are characterized by disruption of the anterior thyroid border and extension of nodular contents into the anterior extrathyroidal area, which may invade the strap muscles. Anterior nodule ruptures are the most common classification subtype (2, 6); experts propose that the relative reduction in pressure exerted on the anterior capsule by the strap muscles compared to the trachea, vasculature, and paraspinal muscles makes the anterior capsule more vulnerable to rupture (6). Nodular contents can result in sinus track formation to the skin, possibly following the artificial track made by the RF needle (**Figure 7**). Dou et al. hypothesized fistula formation as a consequence of nodule rupture from microwave ablation (MWA) due to necrotic breakdown of the needle tract (7).

Medial nodule ruptures occur due to loss of integrity of the medial aspect of the thyroid capsule with ablated material protruding into the potential space between the thyroid and trachea, potentially leading to mass effect on trachea. One patient with reported medial rupture presented with cough due to tracheal irritation from ruptured nodule contents (2). Posterolateral nodule ruptures occur from disruption of the thyroid capsule posterior to the vascular compartment and occurs at the same frequency (6.5%) as medial ruptures (**Table 1**).

### 4.2 Etiology and risk factors

The pathophysiology of nodule rupture is not known; however, several mechanisms have been proposed. Baek et al. described a series of three patients with nodule rupture and proposed acute volume expansion of nodule due to hemorrhage (5). Supporting this theory, Shin et al. in 2011 reported that nodule ruptures demonstrated hyperechoic areas of high attenuation consistent with intranodular bleeding on CT scan. These authors hypothesized that microvessel leakage within the nodule leads to a delayed volume expansion with subsequent rupture (6).

However, in 2021, Chen et al. reported that aspirated nodule rupture contents consisted of mucus and turbid content without evidence of bleeding most consistent with necrotic fluid collection (1). Dou et al. proposed the concept of symptomatic aseptic necrosis (SAN) in which microwave thermal destruction of nodule vasculature resulted in delayed clearance of necrotic content leading to symptomatic irritation of the nodule capsule and surrounding thyroid tissue, a prodrome of impending rupture (7). We suggest that the currently reported case demonstrated a prodrome consistent with SAN on day 7 post-RFA, with ultimate manifestation in nodule rupture at day 30 post-procedure.

Trauma to the treated nodule is another possible mechanism of nodule rupture (5, 6). Kim et al. reported a case of nodule rupture in a patient who habitually massaged his treated nodule leading to diagnosis of rupture at day 60 post-RFA (9). The patient presented in the current study experienced neck extension during dental procedure, a possible contributing factor to the rupture.

The timing of nodule rupture is not predictive of underlying etiology. Nodules with imaging findings consistent with intranodular bleed presented on days 22, 30, and 60 post-RFA (6). The patient with history of habitual massage of treated nodule presented on day 60 post-RFA. Our patient and the patient reported by Chen et al. both presented with extravasation of poorly absorbed necrotic nodule contents, in a similar time frame of 30 and 21, respectively, demonstrating significant overlap of presentation timing of all suspected etiologies (1).

Clinically relevant risks for nodule rupture after RFA have not been delineated. Dou et al. reported increased risk of rupture in larger nodules and in men after microwave ablation. Initial nodule volume and increasing amount of energy used did not reach significance for nodule rupture risk (7). A systematic review by Chen et al. in 2021 speculates that nodule rupture risk following RFA is associated with greater total energy used, severe tissue edema, and increased volumes of necrotic tissue (1). Expert opinion also postulated higher energy deposition near the affected capsule, trauma from multiple RF needle entry points or repetitive patient swallowing during RFA, overlap of energy deposition at capsule entry of the RF needle, and increasing cystic composition of the treated nodule as risk factors for the development of nodule rupture.

### 4.3 Management

Reported management ranges from active surveillance to lobectomy with or without use of antibiotics and analgesia (**Table 2**). Selecting the correct therapy is based on early identification of nodule rupture to prevent unnecessary procedures for diagnosis and management, such as aspiration and empiric antibiotic use (2). Of the currently reported cases, 59.4% were managed with antibiotics, monotherapy, or in combination with additional strategies. However, there are no reported cases of culture-positive rupture fluid collection, suggesting that selective use antibiotics may be the most appropriate management.

**Table 2:** Management of nodule rupture after radiofrequency ablation (RFA).

Management Type	% Patients (N=32)
*Non-Invasive Management*	*56.3% (N=18)*
• Observation (+/− NSAID) Only	31.3% (N=10)
• Antibiotics monotherapy	25.0% (N=8)
• Antibiotics used	59.4% (N=19)
*Invasive Management*	*43.8% (N=14)*
• Aspiration	9.4% (N=3)
• Incision and Drainage	21.9% (N=7)
• Surgical Debridement	9.4% (N=3)
• Lobectomy	3.1% (N=1)

*Additional treatment modalities include NSAID use, aspiration, I&D, surgical debridement.

Impingement on surrounding structures including tracheal compromise, severe dysphagia, or recurrent laryngeal nerve dysfunction may necessitate invasive management like aspiration, I&D, or lobectomy. Aspiration of necrotic content has also been proposed as a method to reduce patient symptomatology but currently lacks evidence of efficacy. Factors that may predict the need for invasive management after nodule rupture have been proposed and include initial nodule diameter and ablative time. Initial diameter of thyroid nodule >4.5 cm has a reported sensitivity and specificity of 69% and 79%, respectively, for predicting the need for invasive management after rupture (1). Shin et al. found that all patients with nodule rupture with an initial greatest diameter >5 cm required invasive management (6). Chen et al. reports RUP, defined as the ablation duration of the RFA session immediately prior to rupture, >20 min is associated with increased odds of requiring invasive treatment of the management of nodule rupture compared to those that can be conservatively managed (OR 1.11, p=0.025). The authors note this association to be of limited clinical utility as RFA procedures commonly exceed 20 min (1).

### 4.4 Outcomes

Nodule rupture can be an unsettling complication to providers and patients alike, requiring serial evaluations and patient discomfort and cosmetic defect. However, all current studies suggest that after management, regardless of the treatment method, all patients experience resolution of symptoms without permanent sequalae (1–4, 6, 10). Theoretic concerns for sequalae include the creation of adhesions that complicate subsequent surgical interventions and risk of extrathyroidal extension of subsequently developed thyroid tumors.

The timeline to resolution of nodule rupture clinical symptoms and imaging features is variable and overall underreported. Che et al. reports symptomatic recovery in 2 weeks following rupture with no intervention (4). Baek et al. described spontaneous reabsorption over 3–6 months (5). In the reported case, symptomatic improvement of pain occurred within 3 days with a slow resolution of pressure and cosmetic defect over 4 months, correlating with the resolution of ultrasonographic features of rupture (**Figure 5**).

## 5 Summary

**Figure 8:**
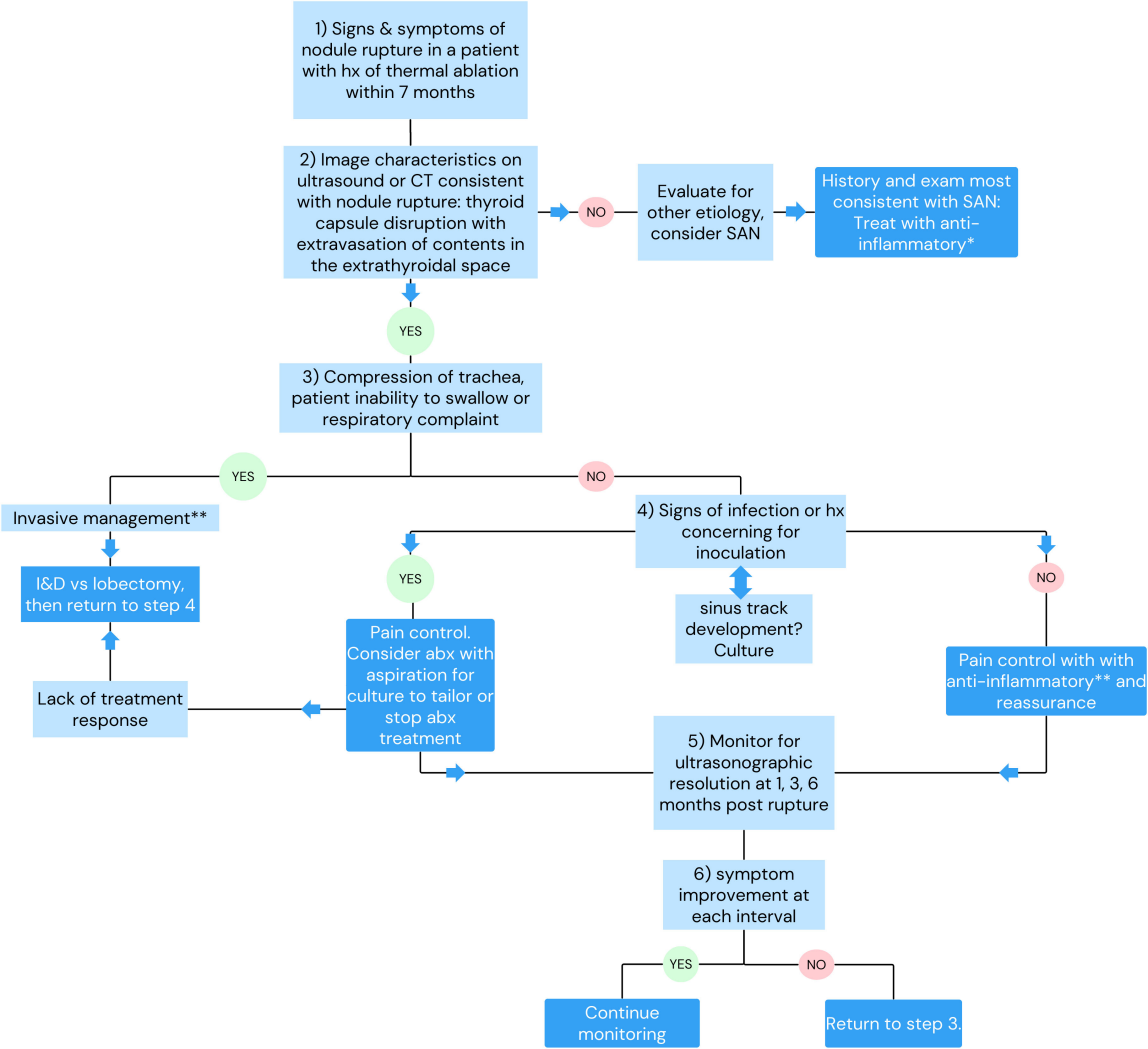
Proposed treatment algorithm for nodule rupture post-RFA.

Thyroid nodule rupture is a major complication following thermal ablation. The available data supports that the majority of nodule rupture patients can be diagnosed with ultrasound and managed conservatively with pain control with serial ultrasounds documenting resolution. Empiric antibiotics in all cases are not necessary and can be reserved for patients with signs of infection or circumstances that increase risk of infectious inoculation, like dental procedures. Patients can be counseled that symptoms of pain resolve before pressure and cosmetic defects over the course of 3–6 months. Fistula formation with cutaneous drainage may occur with anterior nodule rupture. Rare cases will require invasive management such as lobectomy/I&D due to impingement on critical structures of the neck. Improved understanding of nodule rupture and its management is dependent on the accumulation of clinical experience. The adoption of common terminology and collection of proposed data points (**Table 3**) are necessary to improve our understanding of rupture. The current study is the first, to our knowledge, to summarize the presentation, proposed etiologies, management strategies, and outcomes of thyroid nodule rupture after RFA. Based on the current literature review, we propose a management strategy (**Figure 8**) to guide clinicians whose patients experience nodule rupture.

**Table 3:** Comprehensive data collection points for future cases of post-RFA thyroid nodule rupture.

**Baseline characteristics** Nodule size Nodule content (% solid component) Nodule location Nodule distance from affected capsule Gender Age
**Procedure data** Thermal modality used RUP Number of RF needle punctures Number of RFA treatments Total energy deposition from all treatments Power setting used along anterior capsule Maximum power
**Rupture characteristics** Location of rupture Anterior Posterolateral Medial History of Trauma or aggravating activity Symptoms consistent with SAN Diagnostic imaging technique Additional methods of diagnosis Aspiration Other
**Treatment** Antibiotics Anti-Inflammatory Analgesia Aspiration Cytology Culture I&D Surgical wash out Lobectomy Other
**Outcomes** Complete resolution without sequalae Hypothyroidism Chronic cosmetic deformity Chronic discomfort/functional limitation Time to patient reported improvement of symptoms Time to cosmetic improvement Time to negative VRR From rupture diagnosis From baseline nodule volume Development of sinus track Time to resolution Outcome of culture if performed	

## Author contributions

TF: Writing – review & editing, Writing – original draft, Formal analysis, Data curation. SS: Writing – review & editing, Writing – original draft. SH: Writing – review & editing, Writing – original draft. CB: Writing – review & editing, Writing – original draft, Supervision, Methodology, Formal analysis, Data curation, Conceptualization.
